# New Instrument to Measure Hospital Patient Experiences in Flanders

**DOI:** 10.3390/ijerph14111319

**Published:** 2017-10-30

**Authors:** Luk Bruyneel, Else Tambuyzer, Ellen Coeckelberghs, Dirk De Wachter, Walter Sermeus, Dirk De Ridder, Dirk Ramaekers, Ilse Weeghmans, Kris Vanhaecht

**Affiliations:** 1 KU Leuven Institute for Healthcare Policy, University of Leuven, Kapucijnenvoer 35, 3000 Leuven, Belgium; ellen.coeckelberghs@kuleuven.be (E.C.); walter.sermeus@kuleuven.be (W.S.); dirk.deridder@uzleuven.be (D.D.R.); dirk.ramaekers@kuleuven.be (D.R.); kris.vanhaecht@kuleuven.be (K.V.); 2 Flemish Patient Platform, Groenveldstraat 15, 3001 Heverlee, Belgium; else.tambuyzer@vlaamspatientenplatform.be (E.T.); ilse.weeghmans@vlaamspatientenplatform.be (I.W.); 3 Specialised Care Division, Flemish Agency for Care and Health, Flemish public administration, Koning Albert II Laan 35, 1030 Brussels, Belgium; dirk.dewachter@zorg-en-gezondheid.be

**Keywords:** patient satisfaction, public reporting, pay-for-quality, hospitals, HCAHPS

## Abstract

Implementing a standardized patient experience survey may initiate a process to apply pressure on hospitals to attend to improving patient experiences. In Flanders, Belgium, the Flemish Patient Survey was developed between 2011 and 2015. A preliminary version was developed from a scoping review and patient and expert focus groups, and included 27 items for eight hypothesized dimensions: ‘preparing for hospital stay’, ‘information and communication’, ‘coordination’, ‘respect’, ‘privacy’, ‘safe care’, pain management’, and ‘participation’. Exploratory factor analysis for 1076 patients in 17 hospitals found that the data did not fit the dimensions. Adaptations in item wording and response categories were based on the US Hospital Consumer Assessment of Healthcare Providers and Systems. The revised version showed excellent model fit in 22,143 patients in 37 hospitals. Multiple group analysis pointed to evidence of measurement invariance over time across mode of administration, type of nursing unit, and various patient characteristics. Fostering a collaborative approach thus proved successful in implementing a standardized patient experience survey. The most recent findings (2016) illustrate substandard performance and a need for patient-mix adjustment. The Flemish government developed a dedicated website to make findings publicly available and the federal government currently considers patient experiences in devising a pay-for-quality scheme.

## 1. Introduction

Worldwide, survey instruments have been developed to measure patients’ perspectives in various types of services, including general hospital care [1,2], radiology [3], pediatric services [4], elderly care [5], psychiatry [6] and many others. However, the mere implementation of a patient experience survey does not guarantee quality improvement [7]. Although evidence is ambiguous, in the US public reporting of patients’ perspectives of hospital care has shown to provide an incentive in enhancing and reinforcing quality improvement efforts in hospitals [8]. Recently, the push toward tying payments to quality in the hospital Value Based Purchasing program, in which the patient experience has a weight of 25%, could not be linked with improvements in patient experiences, contrary to public reporting [9].

Benchmarking, public reporting and pay-for-quality mechanisms require standardization of questionnaires across hospitals. Easy to interpret and actionable hospital patient experience scores should be implemented nationally and internationally, which may lead to ongoing communication among hospitals and a search for solutions [2,10]. Although some countries have undergone such a process already, despite the wide usage of patient surveys, nationwide implementation of standardized surveys is still rare [11]. This is a serious deficit, since research in the US and Europe has linked patient experiences to quality deficiencies in hospitals’ structures and processes [12,13,14], and hospital managers acknowledge that healthcare quality improvement and the voice of the patient are undeniably interwoven [15]. Belgium, like many other countries, has sought over the past years to increase insight into the quality of its hospitals through key quality metrics. Until recently, Belgian patients did not have access to accurate and reliable information on the quality of hospital care. Now, there is large consensus on the need to strengthen insight into the quality of hospitals and report this information publicly. In Flanders, the northern part of Belgium, the stakeholder-initiated Flemish Indicator Initiative aims to improve the quality of patient care by facilitating the use of clinical process and outcome indicators. In its first uses in acute hospitals, it only collected indicators on mother and childcare, orthopedics, cardiology, breast cancer, stroke, hospital-wide quality, and patient experiences with care. Now, in addition to acute hospitals, indicators are collected in mental healthcare and residential care [16,17]. Feedback is available to organizations and health care providers in the hope that this supports quality improvement initiatives. In addition, it was agreed that the results would be made public in an aggregated manner and hospitals would be encouraged to communicate their results on their website. In 2014, all partners agreed to develop a central website where findings can be consulted by the public.

Before this initiative, there were many fragmented, locally implemented patient surveys in place that measured either satisfaction or experiences with a wide variety of topics, but which were often insufficiently validated. Implementing a standardized questionnaire for public reporting therefore required a multi-stakeholder partnership turning scattered initiatives into a positive dynamism with the final aim of improving patient experiences. The aim of this study is to describe the development and the extensive validation of the Flemish Patient Survey.

Our findings show that a valid questionnaire is now available and allows comparison of findings over time, across mode of administration, type of nursing unit, and various patient characteristics. However, while strongly recommended, survey mode and patient-mix adjustment has not yet been incorporated. This is desirable to allow fair comparison in current public reporting and to make upcoming pay-for-quality mechanisms more equitable. These or other incentives seem crucial since comparison with scores from US hospitals illustrates that there is an urgent need to steer conversations on improvement.

## 2. Materials and Methods

### 2.1. Development and Validation of the Flemish Patient Survey

From 2011 to 2015, we fostered a collaborative approach that involved hospital administrators, the two largest hospital umbrella organizations, the umbrella patient organization, and researchers with extensive experience in instrument development and measuring patient experiences.

To inform the Flemish Patient Survey (FPS) development and validation process, we conducted a scoping literature search, focus groups, a preliminary field test, and a number of region-wide field tests. The scoping review and focus groups were coordinated by staff members with a scientific background at the Flemish Patient Platform, which is an umbrella organization for 110 patient organizations.

First, the objective of our scoping review was to screen for methodologies and instruments to measure quality from a patient perspective. Compared to a systematic review, a scoping review looks at many different study designs and is less likely to assess the quality of included studies. Also, the stakeholder view is important in the reviewing process [18]. We carried out an electronic search on Medline, Embase, Web of Science, Limo, Cinahl and the Cochrane Library in 2011. Relevant keywords and combinations thereof using ‘AND’ or ‘OR’ were ‘patient reported outcome measures’, ‘PRE(M)(s)’, ‘patient satisfaction’, ‘patient experience’, ‘evaluation’, ‘survey’, ‘questionnaire’, ‘service quality’, ‘patient survey’, ‘patient experience survey’, ‘measuring’, ‘measures’ and ‘psychometrics’. All citations were screened by title and abstract for relevance.

Second, also in 2011, patient and expert focus groups assessed this preliminary version. Patient representatives’ opinions were decisive in selecting the items included in the questionnaire. They were invited by e-mail. Experts were invited from within the authors’ network. Focus groups used an informal consensus methodology. It is important to note that focus group participants identified the US Hospital Consumer Assessment of Healthcare Providers and Systems (HCAHPS) as a useful base for assessing criterion validity. HCAHPS includes six composites measuring several aspects of care (‘communication with nurses’, ‘communication with doctors’, ‘staff responsiveness’, ‘pain management’, ‘communication about medicines’, and ‘discharge information’), two questions about ‘physical environment’, and two ‘global ratings’ [19]. A Dutch translation was available from a previous study that used pre- and post- data collection methods to ensure and assess its validity [20,21].

Third, in 2012, university researchers conducted a preliminary field test in a convenience sample of hospitals from within the university’s network. Patients completed both the preliminary version of the FPS and the full HCAHPS at the time of discharge. The central ethics committee of University Hospitals Leuven approved the study.

Fourth, three measurement occasions of four weeks (2013–2014) using the revised version of the FPS were led by all parties involved. All Flemish hospitals (*n* = 55) were invited to participate. The mode of administration was paper-based or electronic, and patients completed the questionnaire at discharge. In all field tests, patients participated voluntary and received no compensation. Non-respondents were not followed-up.

### 2.2. Latest Findings and Mode and Patient-Mix Adjustment

We first describe the current measurement protocol and most recent findings for the year 2016 for the full sample of participating hospitals.

Second, to assess fairness of current comparisons, we explored the need for mode and patient-mix adjustment using data from the Flemish Hospital Network KU Leuven for the year 2016. This is important because findings have been publicly reported by the Flemish government since mid-2015.

### 2.3. Statistical Analysis

For the development and validation of the FPS, for the data from the preliminary field test we conducted exploratory factor analysis (EFA) to explore the dimensionality of our instrument and confirmatory factor analysis (CFA) to establish the multidimensionality of the HCAHPS. For the data from the region-wide field test we conducted EFA and multiple group confirmatory factor analysis (MG CFA) [22] to re-assess the dimensionality of our instrument (EFA) and examine longitudinal and cross-group scalar measurement invariance (MG CFA), respectively. Measurement invariance pertains to examining whether the same dimensionality fits the data across groups and over time. Scalar invariance is one of the more stringent forms of measurement invariance and is required to allow comparison of means across groups [23]. The EFA, CFA, and MG CFA models’ fit evaluation was based on Hu and Bentler’s [24] cut-off criteria (EFA and CFA) and Chen’s [25] allowed changes in these fit indices when studying invariance (MG CFA) for the Comparative Fit Index (CFI) [26]; ranges between 0–1; reasonable if >0.90 and very good if >0.95; a change of ≥−0.01 indicates non-invariance and CFI is the main criterion for assessing change), the Tucker-Lewis Index (TLI [27]; ranges between 0–1; reasonable if >0.90 and very good if >0.95), and the Root Mean Square Error of Approximation (RMSEA [28]; ranges between 0–1; reasonable if <0.08 and very good if <0.05; a change of ≥0.015 indicates non-invariance). For EFA we used weighted least squares estimation and oblique (geomin) rotation that allows the factors to be correlated. For CFA and MG CFA we used weighted least squares estimation using delta parameterization. All items were treated as categorical indicators. The default pairwise present approach was used for handling missing data, which implies that all available observations are used to estimate each correlation [29]. All factor analyses were conducted using Mplus Version 7.1 [22].

To evaluate the need for mode and patient-mix adjustment, we used HCAHPS methodology, for which multiple regression models were conducted and top-box scores were dichotomized as in a public reporting scenario, i.e., patients scoring the hospital 9/10 versus 0–8 [30,31]. A sensitivity analysis was conducted for patients scoring the hospital 8/9/10, and for patients scoring the hospital 10, since these cut-points are also reported in the literature [32,33,34,35]. The regression analyses for this paper were generated using SAS software, Version 9.4 of the SAS System for Windows. Copyright ©2016 SAS Institute Inc. SAS and all other SAS Institute Inc. product or service names are registered trademarks or trademarks of SAS Institute Inc., Cary, NC, USA.

## 3. Results

### 3.1. Scoping Review and Focus Groups

Our interpretation of the many available patient experience items and questionnaires in the literature was guided by three studies that both focused on what patients find important and clearly defined underlying dimensions. First, in one of the few cross-country patient experience studies, Coulter and Cleary covered seven domains of experiences: ‘information and education’, ‘coordination of care’, ‘respect for patient preferences’, ‘emotional support’, ‘physical comfort’, ‘involvement of family and friends’, and ‘continuity and transition’ [36]. Second, in the Netherlands, which borders Flanders and shares the same language, in a similar quality indicator development process, nine indicators were defined from a patient perspective [37]: accessibility, communication and information, respect, shared decision-making, expertise, organization of care, continuity, effective and safe care, and transparency of the cost of care. Third, probably the largest effort to systematically measure patient experiences, the US Hospital Consumer Assessment of Healthcare Providers and Systems (HCAHPS), captures eight dimensions: ‘communication with nurses’, ‘communication with doctors’, ‘staff responsiveness’, ‘pain management’, ‘communication about medicines’, ‘discharge information’, ‘cleanliness and quietness’, and ‘global rating’ [1]. Items within these dimensions were listed. Items from six different unpublished questionnaires that were used in Flemish hospitals were added to this list. From these, the Flemish Patient Platform proposed twenty survey items for eight dimensions reflecting ‘preparing for hospital stay’, ‘information and communication’, ‘coordination’, ‘respect’, ‘privacy’, ‘safe care’, pain management’, and ‘participation’. In addition, one open-ended item on general satisfaction was included to encourage patients to provide additional feedback. From these items, a questionnaire was constructed along the course of hospitalization. To make these decisions, the Flemish Patient Platform was informed by three patient workshops they had previously organized: ‘Patient and hospital’ (2005), ‘How do patients make their choice’ (2010), and ‘The right to good quality health care’ (2011).

Focus group participants (17 patients and 12 experts) subsequently changed the wording of a large number of questions, recommended to drop the open-ended question from the survey, and suggested the use of the HCAHPS as a useful base for assessing criterion validity. Participants extensively discussed the importance of caregiver attitude and respect for the patient as well as caregiver competency. Importantly, participants agreed not to include any items about the hotel function of hospitals, which in previous scattered initiatives to assess patient experiences in Flanders always received much attention. The preliminary version of the FPS included 27 items for the same eight dimensions that resulted from the previous phase (Table 1). For the dimension ‘preparing for hospital stay’, completion depends on whether admission was planned or not. As recommended by several experts, also included in the FPS were the two HCAHPS global ratings of the hospital (recommending the hospital, 0–10 rating of the hospital) and demographic questions. A four-point Likert scale (‘disagree’, ’somewhat agree’, ’largely agree’, ’totally agree’) with a ‘not applicable’ option was used for the core questions. 

### 3.2. Field Test of the Preliminary Version of the Flemish Patient Survey

Our first field test was conducted among 1076 patients in 17 hospitals (39 to 103 patients per hospital). Almost six in ten respondents (57.1%) were female, 58.1% were older than 55, 16.2% rated their health as excellent, and 36.5% had completed higher education. EFA resulted as best in reasonable fit for a two-factor solution which omitted items related to ‘Preparing for hospital stay’ (CFI = 0.94; TLI = 0.93; RMSEA = 0.078). Table 1 displays the standardized factor loading pattern. This factor solution did not discriminate between the hypothesized dimensions. The first factor included six items, all related to information and communication. Two items showed overlap between the two factors. The second factor included all remaining items. In search for a solution, three more complex models were fit to assess multidimensionality from different angles: (1) multilevel EFA to account for the clustering of patients within hospitals [38]; (2) bi-factor EFA to allow a general factor in addition to specific factors [39]; and (3) exploratory structural equation modelling to allow cross-loadings between items and non-target factors [40]. These did not bring about a solution. Also problematic was the high percentage of missing values for a number of items. The dimensional structure of the HCAHPS was assessed through Confirmatory Factor Analysis (CFA), which, contrary to the FPS, showed excellent model fit for the seven proposed factors (CFI = 0.98; TLI = 0.97; RMSEA = 0.021).

### 3.3. Revision of the Flemish Patient Survey and Region-Wide Test

As a result, the Flemish Patient Platform in collaboration with the university researchers made the following notable changes to the preliminary FPS: (1) we improved construction of sentences; (2) we omitted ‘not applicable’; (3) we combined items 2 and 3, and items 2 and 4; (4) we changed response categories ‘disagree’, ’somewhat agree’, ’largely agree’, ’totally agree’ to HCAHPS response categories ‘never’, ‘sometimes’, ‘usually’, and ‘always’; (5) we changed wording of several items to be more similar to HCAHPS; and (6) We reorganized items by their hypothesized dimension.

In our region-wide field tests, a total of 22,143 patients in 37 unique Flemish hospitals completed the modified version of the Flemish Patient Survey (Table 2): (1) June 2013: 13 hospitals and 3026 patients; (2) October 2013: 21 hospitals and 6146 patients; (3) March 2014: 25 hospitals and 12,791 patients. Of the 37 hospitals that participated 28 (76%) had 300 patients or more who participated, and only one had fewer than 100 respondents. Participating hospitals were geographically spread across Flanders. Overall, again almost six in ten respondents (56.4%) were female, 60.8% were older than 55, 14.9% rated their health as excellent, and 32.5% had completed higher education. First, the revised version of the FPS showed a much lower percentage of missing values. Second, eight factors that largely corresponded to the hypothesized dimensions were now identified from EFA (Table 2). These were labeled as follows: ‘information about condition’, ‘information about treatment and procedures’, ‘dealing with patients and collaboration between healthcare providers’, ‘privacy’, ‘pain management’, ‘discharge’, ‘safe care’, ‘preparing for hospital stay’. Model fit was excellent (CFI = 0.98; TLI = 0.98; RMSEA = 0.044). Third, moderate changes in these fit indices pointed to evidence of scalar invariance. This means that the different dimensions of patient experiences were conceptualized similarly over time, and did not depend on the mode of administration, nursing unit/ward or any of the included patient characteristics (Table 3).

### 3.4. Latest Findings and Mode and Patient-Mix Adjustment

Patient experiences with care are currently voluntarily measured twice a year in March/April and September/October. Data submission deadlines follow approximately two months later. Patients are predominantly surveyed at the time of discharge, but hospitals may also opt for administration after discharge. Hospitals are required to recruit patients with a sufficient knowledge of Dutch at surgical, medical, geriatric, maternity, and specialized units, to fully adhere to the FPS, and submit data for a minimum of 150 adult patients on each measurement occasion, i.e., 300 yearly. Data are submitted to the Flemish Agency for Care and Health (Flemish public administration). In 2016, 48 of 55 Flemish acute hospitals participated. Findings for the latest measurement occasion, for 31,892 patients from these hospitals, show that 54.9% of patients rate their hospital 9 or 10 (min: 39.0%–max: 69.3%).

To assess mode and patient-mix adjustment, we obtained data for 5885 patients from 18 hospitals of the Flemish Hospital Network KU Leuven (152 to 653 patients per hospital). The top-box rating of 57.6% for patients rating the hospital 9 or 10 (Table 4) closely corresponded to the 54.9% found in the overall sample of hospitals. Table 4 also shows that variability across hospitals in top-box ratings, whichever method of dichotomization was chosen, was large. Variability across hospitals in the distribution of the potential adjustors was also considerable. Age, health status and type of admission show consistency in the direction but not in the magnitude of the effects for the various methods of dichotomization. For example, younger patients rate the hospital less well, and this effect is most pronounced for dichotomization of 9–10 versus 0–8, compared to other methods of dichotomization. Effects of other variables are more ambiguous. For example, electronic survey compared to paper survey mode shows a large significant effect for the dichotomization of 8–10 versus 0–7, but not for other methods of dichotomization. As such, hospital rankings would be impacted differently by the method of calculating top-box scores. However, for the data presented here, the impact of total adjustment would be relatively small.

### 3.5. Discussion

We described the development of a questionnaire to capture patient experiences with care in the acute hospital setting in Flanders, Belgium.

Although patient experiences cannot be straightforwardly compared across cultures [21], it is worth noting that our finding of a top-box rating of 57.6% for patients rating the hospital 9 or 10 is about 17 percentage points lower compared to the average of 72% in the US [41]. There is thus a need to create learning collaboratives about processes that determine the success of improvement efforts. This will be challenging, as none of the Flemish hospitals are positive outliers. The highest 9 or 10 hospital rating is 66%, whereas several US hospitals attain scores of 85% and higher [41]. Another strategy is that, since July 2015, hospital-level patient experience scores for the two global HCAHPS measures included in the FPS are publicly reported (http://www.zorgkwaliteit.be) once a year. The information on the website details why this indicator is important and what patients can do. All data presented are explained in laymen terms. Other quality metrics reported on this website are diagnosis, treatment and 5-year survival of breast cancer, survival after rectal cancer surgery, patient experiences with patient-centered information on the hospital website, and a number of hospital-wide indicators such as medication safety, hand hygiene compliance, patient identification, and use of a safe surgery checklist. On a monthly basis on average about 3000 unique visitors come to this web site. While it is known that patient experiences are most looked at (almost 13,000 unique views per year), it has not been appraised who visits this web site and how they use the data on this web site. A second incentive is that since early 2017 a stakeholder committee started building a new hospital financing framework in which reimbursement is adjusted on the basis of high-value quality metrics. This pay-for-quality scheme is set to be implemented in 2018 and expected to entail metrics on patient experience. As it currently stands, initially only a small portion of hospital payment will be at stake under this scheme, i.e., ±6 million on a total budget for general hospitals of ±6.4 billion. The aim is however to design a mutable payment incentive structure that encourages improvement. A recent systematic review on pay-for-quality found that the largest improvements in patient outcomes were seen in areas where baseline performance was poor [42]. Improvement in poor performing metrics (such as patient experiences in our study) might at first be realized through public reporting and a small-incentive structure, as hospitals may feel incentivized by the novelty of this dual incentive. Further improvement may result from meaningful increases in incentive payments for which hospitals feel compelled to compete. The US study referred to in the introduction, which could not find any evidence pointing to the implementation of Value Based Purchasing having improved patient experiences, mentioned restructuring of payments and stronger incentives as a possible solution [9]. Patient safety expert Ashish Jha recently proposed a radical paradigm shift to overcome the limitations of current incentives. He suggests letting patients decide about high-quality care, and letting their decision directly determine a considerable percentage of the hospital’s reimbursement [43]. Although we would be careful with suggesting that Belgian decision makers experiment with such transformative ideas, we must, through close monitoring, ensure that our efforts will at least translate into some intentional changes to improve experiences. This has not always been the case in other countries going through this process [44,45]. Improvement should not necessarily depend on external incentives. Engaged leaders and clinicians at multiple levels of the organization must take responsibility and become involved, which was shown to be a common trait among high-performing US hospitals of patients’ reports of care [46].

We outline a number of considerations that merit further attention and highlight a number of limitations that may have hampered our questionnaire validation process.

A first limitation of this study is that no response rates were reported. In England for example, it was found that National Health Service (NHS) hospitals with high patient experience survey response rates may indicate efficient hospital administration, and that low performing hospitals may perform worse with higher response rates [47]. In Flanders, anecdotal evidence points to average response rates of about 20–25% and lower response rates for electronic surveys, yet exact figures are unknown.

Second, mode and patient-mix adjustment is important. Regularly-updated adjustment models for mode and patient mix need to be developed to avoid distorted benchmarking values [31], preferably before the next round of public reporting of patient experiences. In the absence of such adjustments we cannot state that variation in top-box ratings as presented above reflects true variation.

Third, there is currently no mechanism to assess whether all completed questionnaires are transferred to the Flemish Agency for Care and Health. Many good practices and approaches of quality assurance are available from other countries having implemented standardized surveys.

Fourth, with pay for quality being a federal matter, there is a clear need to implement a cohesive national methodology, that is, use the same instrument in the French-speaking part of Belgium. A French-language questionnaire has been in use since 2016 in Flemish hospitals that frequently treat French-speaking patients. Measurement invariance across French and Dutch speaking patients is yet to be assessed.

## 4. Conclusions

The Flemish Patient Survey was rigorously developed and has been implemented in almost all hospitals in Flanders. Its setup is partly inspired by the US HCAHPS, with patient representatives confirming its topics, but also emphasizing preparing for hospital stay, patient safety, and patient information. Flemish global hospital ratings are far from US hospital scores. This is not surprising since the Flemish culture of measuring and reporting patient experiences is still in its infancy. These low scores reinforce the case for tying patient experiences to incentives designed to encourage improvement. Working towards the highest scores attainable means that hospital managers, healthcare providers and patients must be made familiar with these measures. Noteworthy turnarounds are possible, but depend on stakeholders steering conversations on how to improve.

## Figures and Tables

**Table 1:** Exploratory factor analysis for the preliminary version of the Flemish Patient Survey.

Item	Response Categories	Hypothesized Dimension	Missing Data Values (%)	Factor Loadings from Exploratory Factor Analysis	
				Information and Communication	General Factor
1. Was your hospital stay planned in advance?	1	--	8.9%	*Screener (response categories: ‘yes’, ‘no)’*	
2. I received useful and sufficient information on how to prepare for this hospital stay.	2	Preparing for hospital stay	37.3%	--	--
3. This information was provided by my GP.	1	Preparing for hospital stay	39.0%	--	--
4. This information was provided by healthcare providers in the hospital.	1	Preparing for hospital stay	33.8%	--	--
5. I understand the information I received about the cost of my stay.	2	Preparing for hospital stay	31.8%	--	--
6. I received sufficient information about the causes of my condition.	2	Information and communication	15.9%	0.909 *	−0.025
7. I received sufficient information about the possible treatment methods for my condition.	2	Information and communication	17.2%	0.956 *	−0.013
8. I received sufficient information about the consequences of my disease.	2	Information and communication	19.3%	0.853 *	0.047
9. Caregivers always told me in advance why a study, treatment or surgery was needed.	2	Information and communication	10.0%	0.705 *	0.293 *
10. Caregivers told me in advance what exactly an examination, treatment or surgery constituted	2	Information and communication	9.9%	0.725 *	0.301 *
11. Caregivers told me in advance what the possible side effects or effects of the examination, treatment or surgery could be.	2	Information and communication	15.5%	0.664 *	0.257 *
12. Nurses explained things in a way I could understand.	2	Information and communication	5.2%	0.424 *	0.526 *
13. Doctors explained things in a way I could understand.	2	Information and communication	3.3%	0.512 *	0.415
14. Hospital staff did not contradict each other.	2	Coordination	9.5%	0.198 *	0.561 *
15. Nurses treated me with courtesy and respect.	2	Respect	2.1%	0.007	0.856 *
16. Doctors treated me with courtesy and respect.	2	Respect	3.4%	0.139 *	0.775 *
17. My privacy was respected during conversations with caregivers.	2	Privacy	1.3%	−0.150 *	0.971 *
18. My privacy was respected during examinations, treatment and care.	2	Privacy	9.9%	−0.194 *	1.018 *
19. I felt safe in the hands of hospital staff.	2	Safe care	4.5%	0.035	0.860 *
20. Before any treatment, examination or surgery began, my identity was checked by asking for my name, first name and date of birth and my identification band (wristband) was checked.	2	Safe care	9.8%	0.106 *	0.528 *
21. Hospital staff always introduced themselves by name and function.	2	Safe care	7.1%	−0.022	0.569 *
22. Caregivers collaborated well.	2	Coordination	4.7%	−0.007	0.835 *
23. Caregivers sufficiently asked about my pain.	2	Pain management	6.7%	−0.015	0.838 *
24. My pain was well controlled.	2	Pain management	12.3%	0.000	0.772 *
25. Caregivers encouraged me to co-decide on the choices of my research, treatment and care (e.g., washing).	2	Participation	18.9%	0.043	0.710 *
26. I could co-decide on the time of discharge.	2	Participation	24.9%	0.068	0.417 *
27. I felt ready to go home.	2	Participation	18.7%	0.113 *	0.586 *
28. I received adequate information on further treatment after my dismissal from the hospital (e.g., lifestyle rules, rest and work, the use of medicines or tools, control agreements, etc.).	2	Information and communication	20.9%	0.206 *	0.628 *

* Factor loadings present the relationship between factors and factor indicators (i.e., questionnaire items), with loadings closer to 1 representing a stronger relationship. Significance (α = 0.05) is indicated by an asterisk. Bold values indicate major factor loadings. Data are from 2012 for 1076 patients in 17 hospitals. 1 yes/no; 2 disagree/somewhat agree/largely agree/totally agree/not applicable.

**Table 2:** Exploratory factor analysis for the modified version of the Flemish Patient Survey.

Item	Response Categories	Item in Preliminary Version (Table 1)	Missing Data Values (%)	Factor Loadings from Exploratory Factor Analysis							
				Information about Condition	Information about Treatment and Procedures	Dealing with Patients and Collaboration between Healthcare Providers	Privacy	Pain Management	Discharge	Safe Care	Preparing for Hospital Stay
1. My hospital stay was planned in advance.	1	1	12.2%	Screener (response categories: ‘planned’, ‘not planned’)							
2. I received useful and sufficient information from my GP on how to prepare for this hospital stay.	1	3	3.7%	−0.042 *	0.054 *	0.109 *	−0.024	0.015	0.030	−0.025	0.695 *
3. I received useful and sufficient information from hospital staff on how to prepare for this hospital stay.	1	4	8.9%	−0.061 *	0.052 *	−0.124 *	0.028	0.107 *	−0.036 *	0.077 *	0.423 *
4. I received information about the cost of my stay in advance.	1	5	8.8%	0.075 *	0.027	−0.053 *	0.056 *	0.032	−0.056 *	0.119 *	0.558 *
5. Hospital staff provided sufficient information about the causes of my condition.	2	6	8.5%	0.885 *	−0.022 *	0.008	0.022	0.003	−0.016	0.017	0.013
6. Hospital staff provided sufficient information about the possible treatment methods for my condition.	2	7	8.4%	0.919 *	0.029 *	0.034 *	0.021 *	0.040 *	-0.010	−0.068 *	−0.009
7. Hospital staff provided sufficient information about the consequences of my disease.	2	8	9.3%	0.822 *	0.085 *	0.004	−0.005	0.009	0.039 *	0.061 *	0.062 *
8. Hospital staff told me in advance what exactly an examination, treatment or surgery constituted.	2	9	4.9%	0.014 *	0.862 *	0.052 *	0.028 *	0.038 *	−0.022 *	−0.051 *	0.018 *
9. Hospital staff told me in advance why a study, treatment or surgery was needed.	2	10	5.3%	−0.029 *	0.956 *	−0.019	0.016 *	0.023 *	−0.032 *	0.029 *	0.014 *
10. Hospital staff told me in advance what the possible side effects or effects of the examination, treatment or surgery could be.	2	11	8.0%	0.169 *	0.670 *	0.001	−0.006	−0.033 *	0.040 *	0.175 *	0.101 *
11. Nurses explained things in a way I could understand.	2	12	3.7%	0.070 *	0.425 *	**0.373**	0.022	0.083 *	0.046 *	−0.004	0.071 *
12. Nurses treated me with courtesy and respect.	2	15	2.0%	−0.083 *	0.127 *	**0.558**	0.124 *	0.205 *	−0.003	−0.060 *	0.004
13. Doctors explained things in a way I could understand.	2	13	2.0%	0.018	0.073 *	**0.711**	−0.091 *	−0.035 *	0.554 *	0.018 *	0.030 *
14. Doctors treated me with courtesy and respect.	2	16	2.2%	−0.057 *	−0.075 *	**0.828**	0.018 *	0.007	0.521 *	−0.044 *	−0.055 *
15. Hospital staff did not contradict each other.	2	14	6.4%	0.077 *	0.111 *	**0.585**	−0.025	−0.067 *	0.052 *	0.117 *	0.048 *
16. Hospital staff collaborated well.	2	22	2.5%	0.044 *	0.030 *	**0.735**	0.125 *	0.030 *	−0.104 *	0.104 *	0.056 *
17. I felt safe in the hands of hospital staff.	2	19	2.0%	0.064 *	0.207 *	**0.613**	0.230 *	0.103 *	−0.127 *	0.051 *	0.043 *
18. Hospital staff respected my privacy during conversations.	2	17	3.3%	0.012	0.019	0.017	**0.924**	−0.009	0.002	0.012	0.001
19. Hospital staff respected my privacy during examinations, treatment and care.	2	18	3.3%	−0.012	−0.002	0.027	**0.898**	0.022 *	0.023 *	0.005	0.000
20. Hospital staff encouraged me to co-decide on the choices of my research, treatment and care (e.g., washing).	2	25	10.4%	0.133 *	0.207 *	0.0450 *	0.275	−0.023 *	0.045 *	0.353 *	0.132 *
21. Hospital staff always introduced themselves by name and function.	2	21	4.2%	0.012 *	0.019	−0.006	0.015	0.129 *	−0.039 *	0.702	0.010
22. Before any treatment, examination or surgery began, hospital staff checked my identity by asking for my name, first name and date of birth and my identification band (wristband) was checked.	2	20	3.2%	−0.135 *	−0.002	0.089 *	0.018	0.320 *	0.006	0.481	-0.012
23. Hospital staff sufficiently asked about my pain.	2	23	3.4%	0.015	0.055 *	0.009	−0.042 *	**0.855**	−0.008	0.124 *	0.001
24. My pain was well controlled.	2	24	5.2%	0.050 *	−0.014	0.042 *	0.038 *	**0.827**	0.031 *	−0.014	−0.005
25. I could co-decide on the time of discharge.	1	26	6.7%	0.051 *	−0.022	−0.048 *	0.069 *	0.101 *	**0.320**	0.192 *	0.313 *
26. I felt ready to go home.	1	27	5.5%	0.016	−0.011	−0.040	0.072 *	0.245 *		−0.007	0.415 *
27. I received adequate information on further treatment after my dismissal from the hospital (e.g., lifestyle rules, rest and work, the use of medicines or tools, control agreements, etc.).	1	28	7.1%	0.115 *	0.114 *	0.024	0.033	0.214 *	**0.379**	0.065	0.369 *

* Factor loadings present the relationship between factors and factor indicators (i.e., questionnaire items), with loadings closer to 1 representing a stronger relationship. Significance (α = 0.05) is indicated by an asterisk. Bold values indicate major loadings. Data are from 2013 to 2014 for 22,143 patients in 37 Flemish hospitals.1 yes/no. 2 never/sometimes/usually/always.

**Table 3:** Measurement invariance for the modified version of the Flemish Patient Survey.

Patient Characteristics	Scalar Invariance		
	RMSEA	CFI	TLI
Gender	0.062	0.975	0.976
Age	0.068	0.972	0.970
Health status	0.060	0.974	0.974
Education	0.060	0.977	0.977
Type of ward	0.062	0.973	0.973
Measurement occasion	0.064	0.975	0.974
Survey mode	0.062	0.977	0.975

RMSEA = Root Mean Square Error of Approximation; TLI = Tucker-Lewis Index; CFI = Comparative Fit Index. Data are from 2013 to 2014 for 22,143 patients in 37 Flemish hospitals. Following groups were included: gender (female, male), age (18–24, 25–34, 35–44, 45–54, 55–64, 65–74, 75–84, 85+), health status (poor, fair, good, excellent), education (lower education, secondary education, higher, non-university education, university education), type of ward (surgical, medical, maternity, specialty service, geriatrics), measurement occasion (13 hospitals and 3206 patients in June 2013, 21 hospitals and 6146 patients in October 2013, 25 hospitals and 12,791 patients in March 2014), survey mode (paper, electronic).

**Table 4:** Mode and patient-mix adjustment to top category percentages.

		Method of Dichotomization		
Mode and Patient-Mix Adjustors	Variation across Hospitals (Min–Max)	8–10 vs. 0–7	9–10 vs. 0–8 (i.e., Public Reporting)	10 vs. 0–9
Average top box % (min-max)	--	87.5% (78.1–94.7%)	57.6% (40.5–67.1%)	23.7% (17.1–30.0%)
Intercept	--	96.28	75.63	48.85
Gender				
Female	44.1–65.8%	0	0	0
Male	34.2–55.9%	2.10 (0.99) *	1.34 (1.48)	−2.31 (1.25)
Age				
18–24	0.5–6.9%	−14.60 (3.37) *	−21.39 (5.03) *	−14.84 (4.26) *
25–34	6.2–18.8%	−7.90 (2.81) *	−13.45 (4.19) *	−9.70 (3.56) *
35–44	5.2–14.8%	−5.39 (2.74) *	−10.71 (4.08) *	−7.65 (3.46) *
45–54	6.8–20.2%	−3.15 (2.55)	−4.30 (3.80)	−6.99 (3.22) *
55–64	11.9–27.1%	−1.80 (2.45)	−0.49 (3.64)	−2.16 (3.09)
65–74	15.7–24.3%	−2.15 (2.37)	−1.65 (3.53)	−2.09 (2.99)
75–84	5.8–21.8%	−0.26 (2.19)	3.35 (3.46)	−0.13 (2.94)
85+	0.5–12.8%	0	0	0
Health status				
Poor	0.5–8.8%	−20.85 (2.87) *	−28.29 (4.28) *	−21.45 (3.63) *
Fair	19.7–45.2%	−10.89 (1.69) *	−25.30 (2.51) *	−22.64 (2.13) *
Good	35.2–63.0%	−4.29 (1.49) *	−15.18 (2.22) *	−15.82 (1.88) *
Excellent	9.6–19.3%	0	0	0
Education				
Lower education	7.4–30.7%	0	0	0
Secondary education	37.0–54.9%	−0.80 (1.43)	−1.02 (2.36)	−7.59 (1.81) *
Higher, non-university education	20.7–41.3%	−0.97 (1.58)	−1.38 (2.14)	−8.12 (2.00) *
University education	2.1–16.1%	−2.45 (2.19)	−1.37 (3.27)	−12.91 (2.77) *
Living situation				
Co-habiting	67.3–88.0%	0	0	0
Alone	10.7–28.4%	−2.70 (1.32) *	−4.41 (1.96) *	0.32 (1.66)
Service flat etc.	0.4–5%	1.39 (3.37)	−9.40 (5.03) *	−6.32 (4.27)
Type of ward				
Surgical	27.0–65.9%	0.03 (1.21)	−0.87 (1.80)	−0.71 (1.52)
Medical	10.7–42.6%	0	0	0
Maternity	2.6–17.8%	7.94 (2.23) *	6.30 (3.32) *	−1.48 (2.81)
Specialty service	0.6–13.2%	−3.07 (2.79)	−11.76 (4.15) *	−8.82 (3.52) *
Geriatrics	1.9–28.9%	−6.83 (2.08) *	−9.20 (3.11) *	−2.03 (2.61)
Type of admission				
Emergency	21.9–57.7%	0	0	0
Elective	42.3–78.1%	3.50 (1.06) *	7.37 (1.58) *	4.87 (1.34) *
Survey mode				
Paper	0–100%	0	0	0
Electronic	0–100%	−7.27 (2.81) *	−2.08 (4.58)	−0.61 (2.50)

* Significance (α = 0.05) is indicated by an asterisk. Data are from the first semester of 2016 for 5885 patients from 18 hospitals of the Flemish Hospital Network KU Leuven.

## Supplementary Materials

Supplementary File 1
