# Supplementary material for: New Instrument to Measure Hospital Patient Experiences in Flanders
Source: Int J Environ Res Public Health. 2017 Oct 30;14(11):1319. doi: 10.3390/ijerph14111319 (PMC5707958; doi:10.3390/ijerph14111319)
Supplement: Supplementary File 1 [file ijerph-14-01319-s001.pdf]

Vlaams Patiëntenplatform

VLAAMSE PATIËNTEN PEILING 2.0

IN TE VULLEN DOOR EEN MEDEWERKER VAN HET ZIEKENHUIS

**Naam ziekenhuis:**		
**Type bed (kenletter):**	☐ C	☐ D
	☐ G	☐ M
	☐ Sp	

**Code vragenlijst:**	
**Wijze van afname:**	☐ Papieren versie
	☐ Elektronische versie
**Ziekenhuisafdeling:**	

IN TE VULLEN DOOR DE PATIËNT ZELF

Indien u moeilijkheden heeft bij het invullen, kan u hulp vragen aan familie of vrienden. Om uw antwoorden niet te laten beïnvloeden, mogen ziekenhuismedewerkers u niet helpen.

**A. Aard van het ziekenhuisverblijf** (kruis één van de mogelijkheden aan)	
1. Was uw ziekenhuisverblijf gepland?	☐ **Nee**, de datum was niet vooraf bepaald.<br>→ Ga naar C. 'Informatieverstrekking over de aandoening'.
	☐ **Ja**, de datum was vooraf bepaald.<br>→ Ga naar B. 'Voorbereiding op opname'.

**B. Voorbereiding op de opname** (deze 3 vragen enkel invullen indien uw opname gepland was)	**Nee**	**Ja**
2. Ik kreeg van mijn verwijzende arts vooraf informatie over hoe ik mij moest voorbereiden op deze opname in het ziekenhuis.		
3. Ik kreeg van de medewerkers van het ziekenhuis vooraf informatie over hoe ik mij moest voorbereiden op deze opname in het ziekenhuis.		
4. Ik kreeg vooraf informatie over de kosten van mijn opname in het ziekenhuis.		

C. Informatieverstrekking over de aandoening (vanaf hier in te vullen door alle patiënten)

Hieronder gaan een aantal vragen over **"de medewerkers"**. Hiermee worden *alle* medewerkers in het ziekenhuis bedoeld, niet alleen het medisch en verpleegkundig personeel, maar ook de kinesitherapeuten, maatschappelijk werkers, poetsdienst, secretariaat, etc.

	**Nooit**	**Soms**	**Meestal**	**Altijd**
5. De medewerkers informeerden mij over de oorzaken van mijn aandoening.				
6. De medewerkers informeerden mij over de mogelijke behandelingswijzen voor mijn aandoening.				
7. De medewerkers informeerden mij over de gevolgen van mijn aandoening.				

Deze enquête geniet auteursrechtelijke bescherming conform de geldende wetgeving. Het Vlaams Patiëntenplatform vzw wil deze bescherming waarborgen. Hiertoe werd de enquête geregistreerd en kreeg ze vaste dagtekening op 12/01/2015.

D. Informatieverstrekking over onderzoek, behandeling of verzorging				
	**Nooit**	**Soms**	**Meestal**	**Altijd**
8. De medewerkers vertelden mij voor een onderzoek, behandeling of verzorging wat er precies zou gebeuren.				
9. De medewerkers vertelden mij voor een onderzoek, behandeling of verzorging waarom iets nodig was.				
10. De medewerkers vertelden mij voor een onderzoek, behandeling of verzorging wat de mogelijke gevolgen kunnen zijn.				
11. De medewerkers spoorden mij aan om mee te beslissen over welk onderzoek, behandeling of verzorging ik kreeg.				
**E. Wijze van omgaan met patiënten en samenwerking tussen zorgverleners**				
	**Nooit**	**Soms**	**Meestal**	**Altijd**
12. De verpleegkundigen gaven mij begrijpelijke uitleg.				
13. De verpleegkundigen waren vriendelijk en beleefd tegen mij.				
14. De artsen gaven mij begrijpelijke uitleg.				
15. De artsen waren vriendelijk en beleefd tegen mij.				
16. De medewerkers gaven gelijkaardige informatie.				
17. De medewerkers werkten goed samen.				
18. Bij de medewerkers voelde ik mij in veilige handen.				
**F. Privacy**				
	**Nooit**	**Soms**	**Meestal**	**Altijd**
19. De medewerkers respecteerden mijn privacy tijdens gesprekken.				
20. De medewerkers respecteerden mijn privacy tijdens onderzoeken, behandeling en verzorging.				
**G. Veilige zorg**				
	**Nooit**	**Soms**	**Meestal**	**Altijd**
21. De medewerkers stelden zich aan mij voor met naam en functie.				
22. De medewerkers controleerden mijn naam, voornaam, geboortedatum en identificatiebandje bij onderzoeken, behandeling en verzorging.				
**H. Pijn**				
	**Nooit**	**Soms**	**Meestal**	**Altijd**
23. De medewerkers vroegen voldoende naar mijn pijn.				
24. Mijn pijn werd goed onder controle gehouden.				

I. Ontslag uit het ziekenhuis		
	**Nee**	**Ja**
25. Ik kon mee beslissen over het moment van mijn ontslag.		
26. Ik voelde me klaar om naar huis te gaan.		
27. Ik kreeg informatie over de verdere behandeling na mijn ontslag uit het ziekenhuis (bv. over regels voor levensstijl, rust en werken, het gebruik van medicijnen of hulpmiddelen, controle-afspraken, …).		

Deze enquête geniet auteursrechtelijke bescherming conform de geldende wetgeving. Het Vlaams Patiëntenplatform vzw wil deze bescherming waarborgen. Hiertoe werd de enquête geregistreerd en kreeg ze vaste dagtekening op 12/01/2015.

J. Algemene beoordeling

28. Welk cijfer zou u dit ziekenhuis geven voor deze opname, als 0 gelijk is aan het slechtst mogelijke en 10 gelijk is aan het best mogelijke ziekenhuis?										
☐ 0	☐ 1	☐ 2	☐ 3	☐ 4	☐ 5	☐ 6	☐ 7	☐ 8	☐ 9	☐ 10

	Zeker niet	Waar-schijnlijk niet	Waar-schijnlijk wel	Zeker wel
29. Zou u dit ziekenhuis aanbevelen aan uw vrienden en familie?				

K. Enkele vragen over uzelf

Geslacht:	
	☐ Vrouw
	☐ Man

Woonsituatie:	
	☐ Ik woon alleen
	☐ Ik woon samen met mijn partner, familie of vrienden
	☐ Ik woon in een serviceflat, begeleid wonen, rustoord, …

Leeftijd:	
	☐ -18 jaar
	☐ 18 - 24 jaar
	☐ 25 - 34 jaar
	☐ 35 - 44 jaar
	☐ 45 - 54 jaar
	☐ 55 - 64 jaar
	☐ 65 - 74 jaar
	☐ 75 - 84 jaar
	☐ 85+ jaar

Provincie:		
	☐ Oost-Vlaanderen	☐ Henegouwen
	☐ West-Vlaanderen	☐ Namen
	☐ Antwerpen	☐ Luik
	☐ Limburg	☐ Luxemburg
	☐ Vlaams-Brabant	☐ Waals-Brabant
	☐ Brussels Hoofdstedelijk Gewest	☐ Buitenland

**Hoe zou u in het algemeen uw gezondheidstoestand beoordelen?**	☐ Slecht	☐ Redelijk	☐ Goed	☐ Zeer goed

**Wat is het hoogste opleidingsniveau dat u voltooid hebt?** (opleiding afgerond met diploma of getuigschrift)	☐ Lager onderwijs	☐ Secundair onderwijs	☐ Hoger, niet universitair onderwijs	☐ Universitair onderwijs

	Ja	Neen	
**Hebt u de Belgische nationaliteit?**		Zo niet, vul hier uw nationaliteit in:	
**Bent u in België geboren?**		Zo niet, vul hier uw land van herkomst in:	
**Welke taal spreekt u meestal thuis?**	☐ Nederlands	☐ Frans	☐ Duits
	☐ Ander, namelijk:		

Deze enquête geniet auteursrechtelijke bescherming conform de geldende wetgeving. Het Vlaams Patiëntenplatform vzw wil deze bescherming waarborgen. Hiertoe werd de enquête geregistreerd en kreeg ze vaste dagtekening op 12/01/2015.
